# Supplementary material for: Restoration of Corneal Transparency by Mesenchymal Stem Cells
Source: Stem Cell Reports. 2016 Sep 29;7(4):583–90. doi: 10.1016/j.stemcr.2016.09.001 (PMC5063582; doi:10.1016/j.stemcr.2016.09.001)
Supplement: Document S1. Figures S1–S3 [file mmc1.pdf]

Stem Cell Reports, Volume *7*

Supplemental Information

Restoration of Corneal Transparency by Mesenchymal Stem Cells

Sharad K. Mittal, Masahiro Omoto, Afsaneh Amouzegar, Anuradha Sahu, Alexandra Rezazadeh, Kishore R. Katikireddy, Dhvanit I. Shah, Srikant K. Sahu, and Sunil K. Chauhan

Supplemental Data

<u>Figure S1</u>.

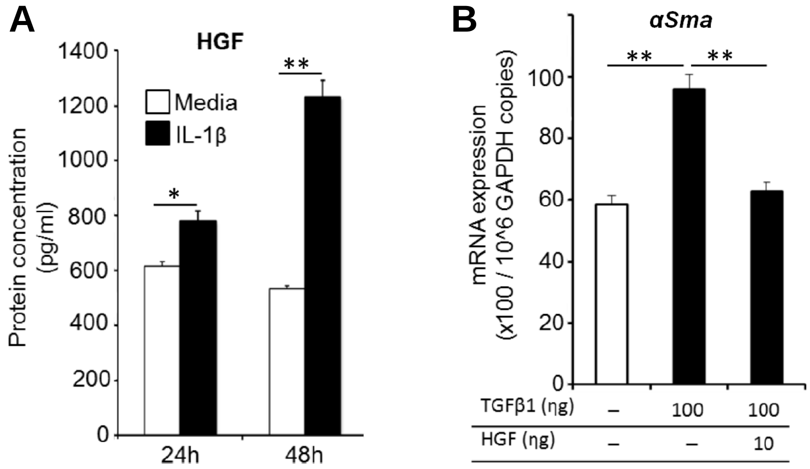

<u>Fig S1</u>: *HGF expressoin by human MSCs, and effect of recombinant HGF on human corneal firoblast.* [A]. Human MSCs secrete elevated levels of HGF upon IL1β stimulation. Human MSCs (purchased from Lonza) were cultured in medium alone (white bars) or with IL1β (100ng/ml, Peprotech) (black bars) for 24 hrs and 48 hrs. HGF protein levels were estimated in culture supernatants using ELISA (R&D systems). **[B].** HGF inhibits differentiation of human corneal fibroblasts into myofibroblasts. Human corneal fibroblasts (gifted from James Zieske laboratory, Schepens Eye Reaesch Institute) were stimulated with human recombinant TGFβ1 (100ng/ml, Peprotech) in the presence or absence of rhHGF (10ng/ml, R&D Systems) for 24hrs. *α-SMA* expression (normalized to internal control *GAPDH*) was evaluated using real-time PCR.The values shown are mean ± SD (error bars) from three independent experiments performed in triplicates, * $p<0.02$, ** $p<0.001$.

<u>Figure S2</u>.

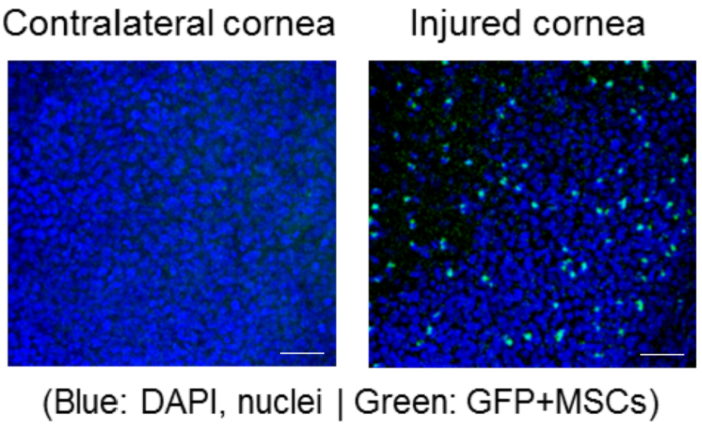

<u>Fig S2:</u> ***MSCs selectively home to injured corneas.*** Corneal injury was induced by mechanical removal of corneal epithelium and anterior stroma in C57BL/6 mice. After 1 hour of injury, GFP-expressing MSCs purified from GFP transgenic C57BL/6 mice (Jackson Laboratory, Bar Harbor, ME, USA) were intravenously administered to mice (n=5) and corneas were harvested at 7 day post injury. Corneas were fixed with 4% paraformaldehyde for 20 minutes and permeabilized with 0.5% TritonX-100 for 10 minutes, and then immunostained with Alexa Fluor 488-conjugated anti-GFP (Invitrogen). Corneas were then mounted onto slides with DAPI containing mounting medium (Vector Laboratories, Burlingame, CA). Representative confocal micrographs of whole mount corneas showing the presence of GFP+ MSCs (Green color) in the injured cornea, but not in the contralateral (uninjured) cornea (scale bar 100µm).

Figure S3.

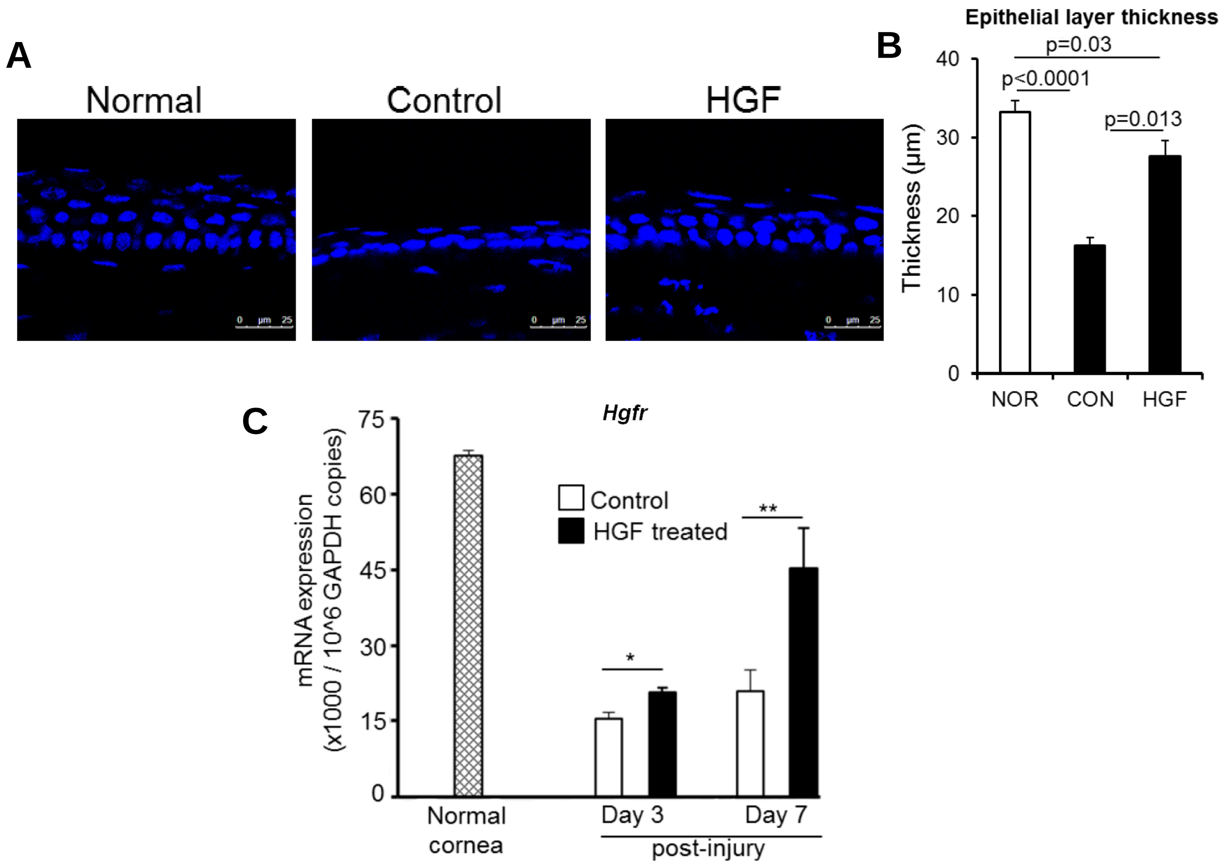

Fig S3: ***HGF augments epethelial stratification and HGF-R (c-met) expression in the cornea after injury.*** **[A]** At 7 days post injury, corneas were harvested from normal, albumin-treated and HGF-treated mice. Corneal cross sections were stained with the nuclear stain DAPI to visualize corneal epithelial cell layer using confocal microscope (scale bar: 25µm). **[B]**. Bar chart showing the thickness (µm) of the epithelial cell layer in normal (white bar), control-injured and HGF-treated injured corneas (black bar). **[C]**. At 3 and 7 days post injury, corneas were harvested from normal (checked bar), mouse albumin-treated control injured (white bar) and HGF-treated (black bar) injured groups. Total RNA was isolated from harvested corneas. *Hgf-R* mRNA expression was quantitated using real-time PCR. *Gapdh* was used as a internal control.The values shown are the mean ± SD; n=5-6 mice/group.
