# Supplementary material for: Restoration of Corneal Transparency by Mesenchymal Stem Cells
Source: Stem Cell Reports. 2016 Sep 29;7(4):583–90. doi: 10.1016/j.stemcr.2016.09.001 (PMC5063582; doi:10.1016/j.stemcr.2016.09.001)
Supplement: Document S2. Article plus Supplemental Information [file mmc2.pdf]

Stem Cell Reports
Report
OPEN ACCESS

Restoration of Corneal Transparency by Mesenchymal Stem Cells

Sharad K. Mittal,[1,2,6] Masahiro Omoto,[1,3,6] Afsaneh Amouzegar,[1,2] Anuradha Sahu,[1] Alexandra Rezazadeh,[1] Kishore R. Katikireddy,[1,2] Dhvanit I. Shah,[4] Srikant K. Sahu,[1,5] and Sunil K. Chauhan[1,2,*]
[1]Schepens Eye Research Institute, Massachusetts Eye and Ear, 20 Staniford Street, Boston, MA 02114, USA
[2]Department of Ophthalmology, Harvard Medical School, Boston, MA 02114, USA
[3]Department of Ophthalmology, Keio University School of Medicine, Tokyo 160-8582, Japan
[4]Brigham and Women's Hospital, Harvard Medical School, Boston, MA 02114, USA
[5]L.V. Prasad Eye Institute, Bhubaneswar, Odisha 751024, India
[6]Co-first author
*Correspondence: sunil_chauhan@meei.harvard.edu
http://dx.doi.org/10.1016/j.stemcr.2016.09.001

SUMMARY

Transparency of the cornea is indispensable for optimal vision. Ocular trauma is a leading cause of corneal opacity, leading to 25 million cases of blindness annually. Recently, mesenchymal stem cells (MSCs) have gained prominence due to their inflammation-suppressing and tissue repair functions. Here, we investigate the potential of MSCs to restore corneal transparency following ocular injury. Using an in vivo mouse model of ocular injury, we report that MSCs have the capacity to restore corneal transparency by secreting high levels of hepatocyte growth factor (HGF). Interestingly, our data also show that HGF alone can restore corneal transparency, an observation that has translational implications for the development of HGF-based therapy.

INTRODUCTION

A transparent cornea is crucial for optimal vision. Ocular trauma, a leading cause of loss of corneal transparency, accounts for approximately 25 million cases of blindness annually (Resnikoff et al., 2008; Whitcher et al., 2001). During ocular injury, inflammation-induced transforming growth factor β (TGF-β), particularly TGF-β1 and TGF-β2, drive the differentiation of corneal fibroblasts (activated keratocytes) into α-smooth muscle actin (αSMA)-expressing myofibroblasts (Jester et al., 1997; Torricelli et al., 2016), which are themselves opaque and produce disorganized extracellular matrix, leading to the development of corneal opacity and scarring (Jester, 2008; Jester et al., 2012; Ljubimov and Saghizadeh, 2015). Recently, mesenchymal stem cells (MSCs) have been linked to a variety of anti-inflammatory and repair functions in both ocular and non-ocular tissue injuries (Basu et al., 2014; Jiang et al., 2002; Lan et al., 2012; Lee et al., 2014; Uccelli et al., 2008; Wang et al., 2011). However, ocular injuries involving the cornea undergo a wound-healing process that often results in scar formation and loss of corneal transparency. Here, we report that bone marrow-derived MSCs are capable of restoring corneal transparency after injury involving corneal stroma. Specifically, we show that MSCs secrete high levels of hepatocyte growth factor (HGF), which inhibits the generation of opacity-inducing myofibroblasts. Furthermore, we show that HGF alone can restore corneal transparency in an in vivo model of eye injury, a finding that offers an HGF-based therapeutic approach that could potentially eliminate the need for cell-based and conventional therapies.

RESULTS AND DISCUSSION

Inflammatory Milieu Drives MSCs to Secrete Elevated Levels of HGF

The cornea is the most anterior tissue of the eye that comprises the epithelium, stroma, and endothelium (Nishida and Saika, 2011). Ocular injuries involving the stroma (Figure 1A) lead to corneal scarring and compromised vision (Jester, 2008; Whitcher et al., 2001). The aim of this study was to determine whether MSCs have the potential to restore corneal transparency following injury. To investigate this, we first screened MSCs for expression of potential anti-inflammatory and growth factors under both homeostasis and inflammatory conditions. In vitro expanded and functionally characterized bone marrow-derived MSCs (Figures 1B and 1C) were cultured in the absence (medium alone) or presence of interleukin-1β (IL-1β) (to mimic injury-induced inflammatory milieu) for 24 hr, followed by quantification of tumor necrosis factor-stimulated gene 6 (*Tsg-6*), *Il-10*, *Tgf-β1*, and *Hgf* transcripts using real-time qPCR (Figure 1D). Strikingly, IL-1β stimulation greatly enhanced the expression of *Hgf* in MSCs compared with unstimulated cells. In contrast, *Tgf-β1* expression was significantly reduced in IL-1β-stimulated MSCs. The steady-state expression of *Tsg-6* was moderately increased, and *Il-10* remained unchanged upon IL-1β stimulation. In addition, ELISA performed on culture supernatants corroborated the qPCR data and showed a 2.5-fold increase in HGF secretion by IL1β-stimulated MSCs (Figure 1E). These in vitro data demonstrate that MSCs express high levels of HGF in an inflamed environment. We also confirmed these findings using human MSCs. Our data showed that human

This is an open access article under the CC BY license (http://creativecommons.org/licenses/by/4.0/).

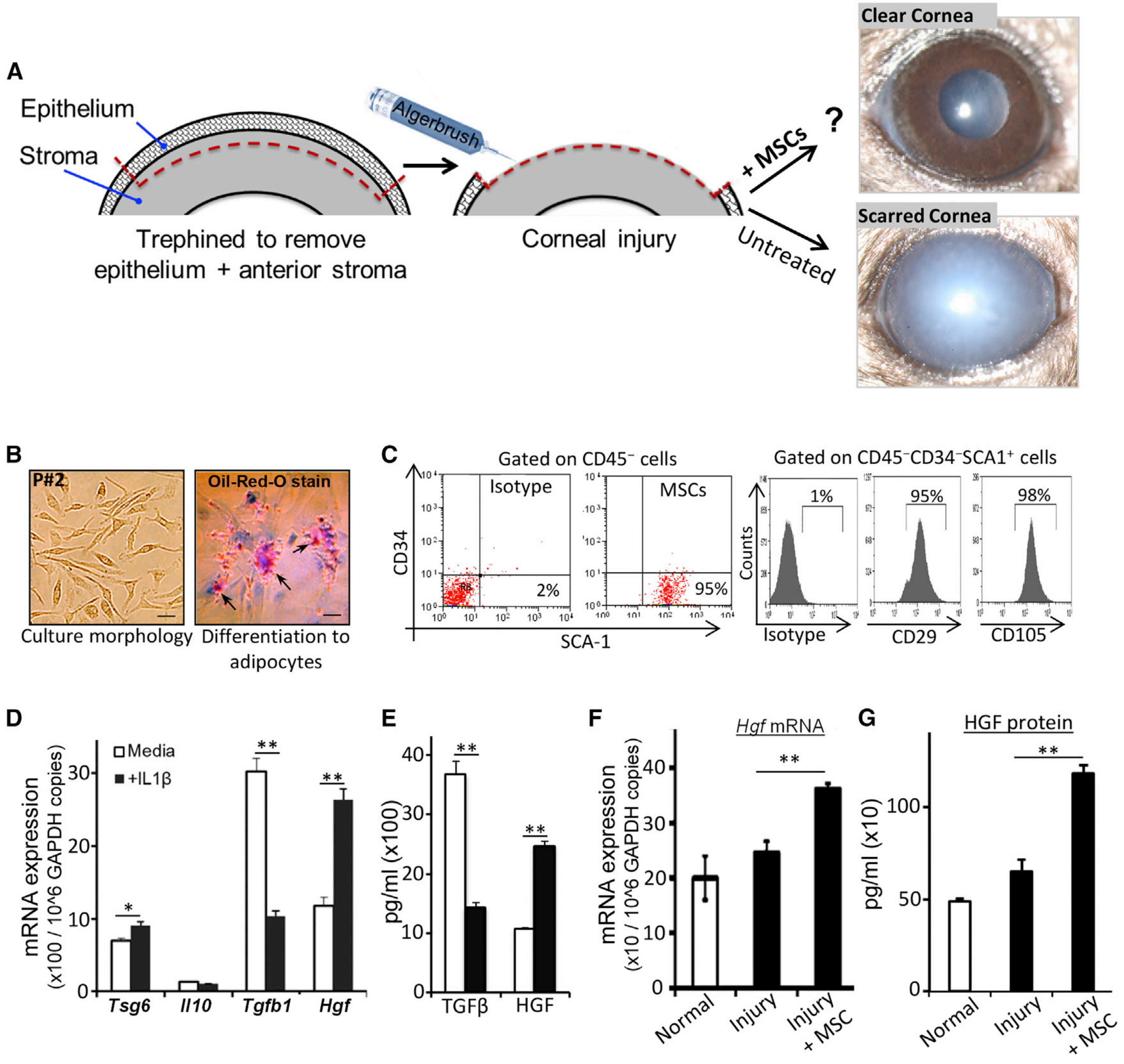

Figure 1. MSCs Secrete High Levels of HGF upon Stimulation with IL-1β

(A) Schematic showing injury model of mouse cornea created by mechanical removal of epithelium and anterior stroma, and effect of mesenchymal stem cell (MSC) administration on corneal opacity.

(B) Micrographs showing MSC morphology in culture at second passage, and differentiation of MSCs into adipocytes. MSCs were cultured in adipogenic medium for 2 weeks and stained with oil red O dye; red-colored vacuoles (arrows) were observed within the cytoplasm, indicating their differentiation into adipocytes. Scale bar, 25 μm.

(C) Phenotypic characterization of in vitro expanded MSCs using flow cytometry confirmed their surface phenotype of $CD45^{-}CD34^{-}SCA1^{+}CD29^{+}CD105^{+}$ cells.

(D) MSCs were cultured in medium alone or with IL-1β for 24 hr. mRNA expression of indicated genes in MSCs were analyzed using real-time PCR.

(E) Protein expression of TGF-β1 and HGF was confirmed in culture supernatants of MSCs cultured in the presence or absence of IL-1β for 24 hr using ELISA. The values of mRNA and protein expression are shown as mean ± SD of three independent experiments.

(F and G) In vitro expanded MSCs were intravenously injected into the C57BL/6 mice 1 hr after corneal injury. Healthy corneas without injury were used as normal control. Corneas were harvested after 3 days, and (F) mRNA and (G) protein expressions of HGF were measured using real-time PCR and ELISA, respectively.

The values shown are mean ± SD and each corneal injury group consists of n = 6 mice. $^{*}p < 0.003$, $^{**}p < 0.0001$.

bone marrow-derived MSCs constitutively expressed high levels of HGF, which was significantly upregulated upon stimulation with recombinant human IL-1β (Figure S1A).

To determine whether in vivo administration of MSCs leads to high levels of HGF at inflamed injury site, we utilized a well-characterized sterile injury model of mouse

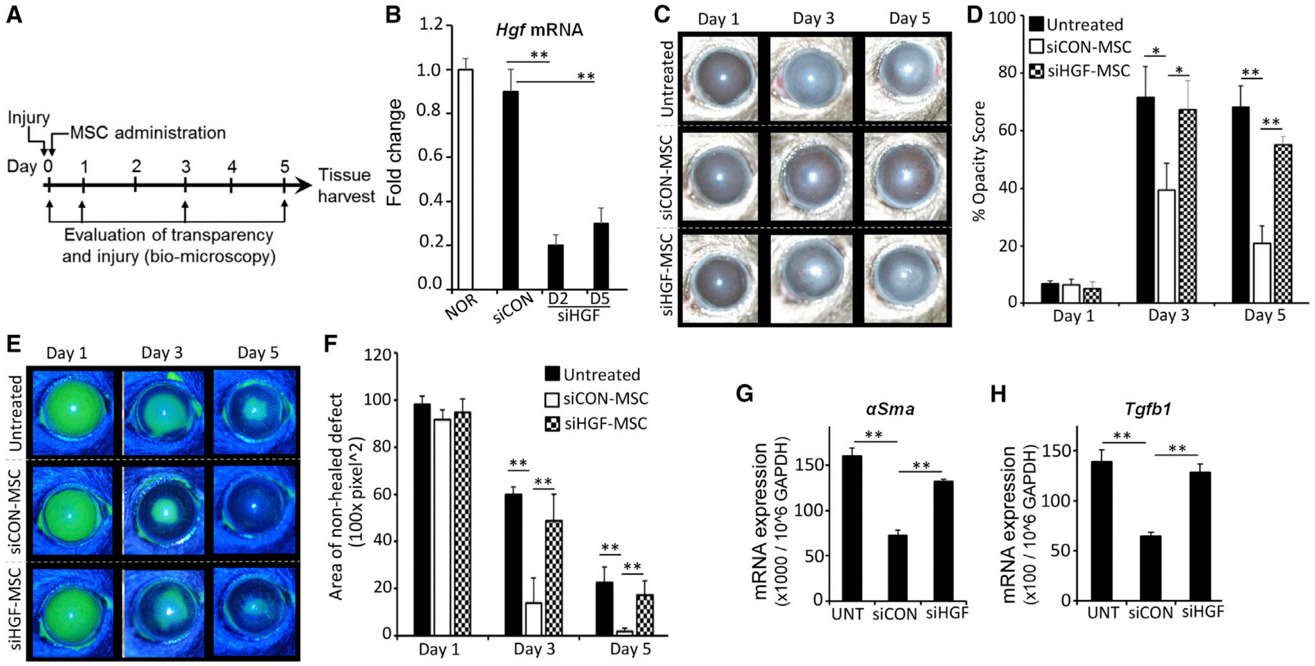

Figure 2. Restoration of Corneal Transparency Is Dependent upon HGF Expression by MSCs

(A) Schematic of experimental design.

(B) Real-time PCR analysis showing efficacy of *Hgf*-specific siRNA (siHGF) versus control siRNA (siCON) on downregulation of HGF expression in mesenchymal stem cells (MSCs). After corneal injury was induced in C57BL/6 mice, MSCs treated with control or *Hgf*-specific siRNA were intravenously administered 1 hr post injury and followed for 5 days. At days 1, 3, and 5 post injury, photographs of injured cornea with or without green fluorescein stain were captured using slit-lamp biomicroscopy. Corneal fluorescein staining was used to indicate epithelial defects and bright-field micrographs were used to evaluate corneal opacity.

(C and D) Representative bright-field microscopic images of injured cornea (C) were quantitated using Image J software to measure the corneal opacity scores (D).

(E) Representative biomicroscopic images showing green fluorescein-stained injured cornea.

(F) The fluorescein-stained area was quantitated using ImageJ software. A smaller area of fluorescein staining represents faster repair of corneal injury.

(G and H) At day 5 post injury, corneas were harvested. Total RNA was isolated from harvested corneas, and real-time PCR was performed to analyze mRNA expression of (G) *α-Sma* and (H) *Tgf-β1*.

The values shown are mean ± SD and each corneal injury group consists of n = 6 mice. $^*p < 0.02$, $^{**}p < 0.005$.

cornea (Basu et al., 2014; Hutcheon et al., 2007). Injury was induced by mechanical removal of corneal epithelium and anterior stroma (Figure 1A); 1 hr after injury, MSCs (5×10^5/0.1 mL/mouse) were intravenously injected in mice. Using GFP-expressing MSCs (Figure S2), we additionally confirmed that MSCs specifically home to the injured eye (Lan et al., 2012; Omoto et al., 2014). Normal corneas without injury and corneas with injury alone (without MSC administration) served as controls. On day 3 after injury, corneas were harvested, and qPCR and ELISA were performed to measure HGF levels. Indeed, injured corneas from MSC-injected mice showed significantly higher levels of HGF at both transcript (Figure 1F) and protein (Figure 1G) levels compared with injured and normal corneas.

Capacity of MSCs to Restore Corneal Transparency Is Dependent upon Their HGF Expression

Based on our in vivo data and because previous reports have ascribed an anti-fibrotic function for HGF (Herrero-Fresneda et al., 2006), we hypothesized that HGF could be a putative MSC-expressed factor that could contribute to the restoration of transparency in injured corneas. We therefore determined whether altering HGF expression within MSCs influenced opacity in a sterile injury model of mouse cornea (Figures 1A and 2A). HGF expression in MSCs was knocked down using small interfering RNA (siRNA) (Abed et al., 2015), which led to nearly 80% reduction of *Hgf* expression compared with control siRNA (Figure 2B). MSCs transfected with *Hgf* siRNA or control siRNA were pre-stimulated with IL-1β for 6 hr, then intravenously

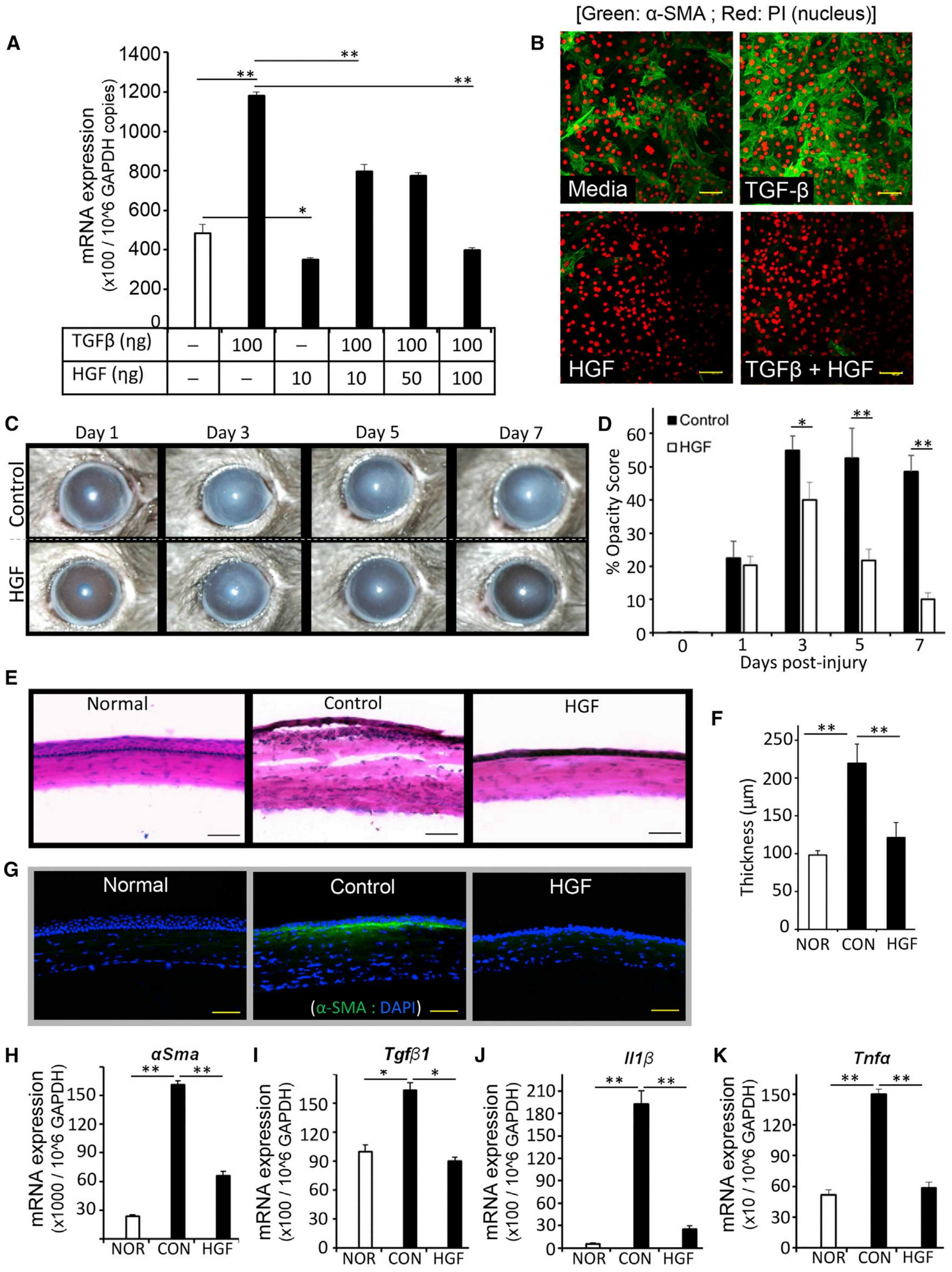

Figure 3. HGF Alone Is Sufficient to Inhibit Corneal Opacity and Inflammation

(A and B) A corneal fibroblast cell line (MK/T1) was stimulated with TGF-β1 in the presence or absence of HGF for 24 hr. α-SMA expression was assessed (A) at mRNA level using real-time PCR and (B) at protein level by immunohistochemistry. The values shown are the mean ± SD of three independent experiments.

(legend continued on next page)

administered to the mice 1 hr post injury. Injured corneas with no MSC administration served as untreated controls. Slit-lamp biomicroscopy was used to monitor the extent of corneal opacity and wound healing for 5 days. Corneas of mice injected with control siRNA-treated MSCs showed a significant reduction in corneal opacity at days 3 and 5 post injury compared with corneas from *Hgf* siRNA-treated MSCs and untreated mice (Figures 2C and 2D). To determine the extent of wound repair, we used corneal fluorescein staining to assess the epithelial defect (Figures 2E and 2F). A smaller area of fluorescein (green) represents a faster rate of wound healing. A complete and significantly more rapid wound repair was seen in mice injected with control siRNA-treated MSCs compared with corneas from *Hgf* siRNA-treated MSCs and untreated control mice. Previous reports have shown similar effects of wild-type MSCs on wound repair (Lan et al., 2012; Lee et al., 2014). After 5 days of injury, corneas were harvested to assess expression levels of *α-Sma* and *Tgf-β1* using qPCR. Data showed a markedly decreased expression of *α-Sma* and its inducer cytokine *Tgf-β* (Yi et al., 2014) in the corneas of mice injected with control siRNA-treated MSCs compared with the corneas of *Hgf* siRNA-treated MSCs and untreated mice (Figures 2G and 2H). These data clearly demonstrate that HGF expression by MSCs is crucial for inhibiting the expression of opacity-inducing α-SMA and TGF-β, and restoring corneal transparency in the injured eye.

Topical Administration of HGF Alone Is Sufficient to Restore Corneal Transparency in Ocular Injury

Finally, the functional and translational relevance of HGF in restoring corneal transparency was confirmed by investigating the effect of HGF alone (without MSC administration) using both in vitro and in vivo model systems. First, to experimentally address whether HGF can inhibit expression of α-SMA in corneal fibroblasts, we stimulated a well-characterized corneal fibroblast cell line (MK/T1) (Gendron et al., 2001) with TGF-β1 in the absence or presence of recombinant mouse HGF for 24 hr. Unstimulated cultures served as a control. HGF treatment showed a dose-dependent suppression of TGF-β-induced *α-Sma* expression in corneal fibroblasts (Figure 3A). Consistent with our data in mice, we also observed that human recombinant HGF completely suppressed TGF-β1-induced *α-SMA* expression in human corneal fibroblasts (Figure S1B).

We also confirmed the effect of HGF on TGF-β-induced α-SMA protein expression using immunohistochemistry. HGF completely suppressed TGF-β-stimulated α-SMA protein expression in corneal fibroblasts and prevented their conversion to myofibroblasts (α-SMA$^+$ cells: green) (Figure 3B), which are the primary cause of corneal opacity (Jester, 2008; Jiang et al., 2002). Interestingly, HGF treatment (Figures 3A and 3B; media versus HGF) also significantly reduced the baseline expression of α-SMA in corneal fibroblasts, suggesting that HGF alone could be effective in reversing pre-formed myofibroblasts into α-SMA-negative fibroblasts. Using this information, we sought to investigate whether in vivo administration of HGF can suppress corneal opacity. Corneal injury was induced as described above (Figure 1A), 5 μL of 0.1% recombinant mouse HGF or mouse serum albumin (control) was applied topically to the injured eye twice daily for up to 7 days after injury, and slit-lamp biomicroscopy was used to monitor corneal opacity (Figure 3C). At day 3 post injury, both groups showed a significant development of corneal opacity. However, the corneas of HGF-treated mice exhibited a significant reduction in opacity on day 5 and a near complete restoration of transparency on day 7 compared with mouse albumin-treated control corneas (Figure 3D). After 7 days post injury, corneas were harvested to confirm the effect of HGF on injury-induced opacity at cellular and molecular levels. H&E staining of corneal cross-sections revealed normalization of corneal tissue structures only in HGF-treated mice (Figure 3E), whereas albumin-treated control corneas showed a significant increase in tissue thickness accompanied by infiltration of inflammatory cells (Figures 3E and 3F). Moreover, HGF-treated corneas showed increased stratification of the epithelial cell layer (Figures S3A and S3B). Both confocal micrographs of immunostained corneas (Figure 3G) and qPCR (Figure 3H) showed a significant reduction in the expression of α-SMA in HGF-treated corneas compared with control corneas. Moreover, mRNA expression levels of α-SMA-inducer cytokine *Tgf-β1* (Figure 3I), and the inflammatory cytokines *Il-1β* (Figure 3J) and *Tnf-α* (Figure 3K) were significantly reduced in HGF-treated corneas compared with

(C–K) Corneal injury was induced by mechanical removal of corneal epithelium and anterior stroma in C57BL/6 mice. Thereafter, 5 μL of 0.1% murine recombinant HGF in PBS per eye was applied topically to the injured eye twice a day up to 7 days after injury. A control group received a similar dosage of mouse serum albumin. At days 1, 3, 5, and 7 post injury, bright-field photographs of injured corneas were captured to evaluate corneal opacity using slit-lamp biomicroscopy. Representative bright-field images of injured corneas (C) were quantitated using Image J software to assess corneal opacity scores (D). Corneas were harvested at 7 days post injury. Cross-sections were stained with H&E to visualize corneal tissue structure and infiltration of inflammatory cells (E), and measure corneal tissue thickness (F). For immunocytochemistry analysis (G), cross-sections were immunostained with the fibrosis marker α-SMA (green). In addition, harvested corneas were analyzed for their mRNA expression of (H) *α-Sma*, (I) *Tgf-β1*, (J) *Il-1β*, and (K) *Tnf-α* using real-time PCR.
The values shown are mean ± SD and each corneal injury group consists of n = 6 mice. $^*p < 0.01$, $^{**}p < 0.005$. Scale bars, 50 μm.

albumin-treated corneas. The fact that HGF-treated corneas showed high expression of *Hgf-R* (*c-Met*) compared with control corneas (Figure S3C) further supports our finding that HGF signaling inhibits α-SMA expression. Collectively, these findings indicate that HGF administration alone is sufficient to restore transparency in corneal injury by suppressing conversion of corneal fibroblasts into αSMA^{+} myofibroblasts and by inhibiting tissue infiltration of inflammatory cells, which secrete inflammatory cytokines and proteolytic enzymes, leading to degradation and remodeling of the extracellular matrix (Ljubimov and Saghizadeh, 2015).

Conventional treatments for ocular injuries involving corneal scarring vary from topical immunosuppressive steroids to corneal transplantation. However, (1) the increased risk of infection and delayed wound healing, (2) immune rejection of the transplant, and (3) shortage of cornea donors remain major limitations to such treatment (Hamil, 2011). Recently, due to their unique immunomodulatory property, MSCs have been used in experimental and clinical settings to treat a variety of tissue injuries and inflammatory diseases (Basu et al., 2014; Lan et al., 2012; Lee et al., 2014; Uccelli et al., 2008; Wang et al., 2011). Here, we ascribe a hitherto unknown function of MSCs in restoring corneal transparency following ocular injury. We report that MSCs inhibit the expression of opacity-inducing α-SMA and its inducer TGF-β in the injured cornea by secreting HGF. Furthermore, we show that administration of HGF alone can suppress corneal opacity and inflammation. Given that clinical-grade production of cell-based therapies is cost prohibitive, our findings offer the promise of HGF-based modalities for treating ocular conditions that compromise corneal transparency and vision.

EXPERIMENTAL PROCEDURES

Animals

Six- to 8-week-old male C57BL/6 wild-type mice (Charles River Laboratories) were used in these experiments. The protocol was approved by the Schepens Eye Research Institute Animal Care and Use Committee, and all animals were treated according to the ARVO Statement for the Use of Animals in Ophthalmic and Vision Research.

Corneal Injury

Mice were anesthetized and a 3-mm superficial keratectomy was performed as previously described (Basu et al., 2014; Hutcheon et al., 2007). In brief, under a dissecting microscope the central area of the cornea was demarcated with a 3-mm trephine and rotated gently to cut into the stroma. The circular area was traced with a sharp pair of surgical forceps, and the corneal epithelium and basement membrane, including the anterior portion of the stroma, were removed using a hand-held Algerbrush II (Alger Equipment). Following injury, corneas were flushed with sterile saline and subsequently covered with Vetropolycin (bacitracin-neomycin-polymyxin) ophthalmic ointment.

Corneal opacity was determined by taking bright-field images using a biomicroscope. Corneal wounds were monitored by placing 1 μL of 2.5% sodium fluorescein (vital staining) on the ocular surface. After 3 min, the ocular surface was visualized by slit-lamp biomicroscope under cobalt blue light, and digital pictures of corneal defects were captured. Degree of opacity and area of injury (fluorescein-stained green color) were calculated using the NIH ImageJ (version 1.34s) software.

Isolation, Expansion, and Characterization of MSCs

Bone marrow was harvested from femurs of euthanized C57BL/6 mice. MSCs were phenotypically and functionally characterized as per criteria defined by The International Society for Cellular Therapy (Dominici et al., 2006), using the previously described plastic adherence method of MSC cultivation (Lan et al., 2012; Lee et al., 2014), and bone marrow cells were cultured in murine MSC-specific MesenCult medium with supplement (STEMCELL Technologies). Non-adherent cells were removed by changing medium every 2 days, and at passage 2 the MSCs were harvested to be used in experiments. Before using MSCs in indicated experiments, cells were characterized phenotypically for the expression of MSC markers ($CD45^{-}CD34^{-}SCA1^{+}CD29^{+}CD105^{+}$) by flow cytometry and functionally by their in vitro differentiation into adipocytes using MesenCult adipogenic stimulatory supplements (STEMCELL). Oil red O (Sigma-Aldrich) staining was used to confirm the differentiation of MSCs into the adipocytes.

siRNA Transfection

MSCs (1.5×10^{6} cells) were plated in a 75-cm^{2} flask and incubated for 18–24 hr to reach to 60%–70% confluency. The cells were then washed and transfected with 4.8 μg of *Hgf*-specific or non-specific control siRNA duplex using transfection reagent in siRNA transfection medium according to the protocol suggested by the manufacturer (Santa Cruz Biotechnology). After overnight incubation, transfection medium was replaced with normal MSC growth culture medium and cells were cultured for an additional 2 days. Knockdown efficiency of siRNA was validated by real-time PCR using *Hgf*-specific primers after 2 and 5 days of transfection.

MSC or HGF Administration

In vitro expanded wild-type or *Hgf*-silenced MSCs were pre-stimulated with IL-1β for 6 hr, and 5×10^{5} MSCs in 100 μL of normal saline per mouse were injected to mice 1 hr after corneal injury. Mice were placed in a restraining tube without anesthesia and the tail cleaned with 70% ethanol. The tail was pulled gently and cells in 100 μL of PBS were injected into the tail vein. Five microliters of 0.1% murine recombinant HGF protein (R&D Systems) or mouse serum albumin (Sigma-Aldrich) was applied topically to the injured eye twice daily for up to 7 days after injury.

In Vitro MK/T1 Cell Stimulation

The mouse corneal fibroblast cell line MK/T1 (Gendron et al., 2001) was seeded at 1×10^{5} cells per well in 24-well plates and

cultured in medium alone or stimulated with 100 ng/mL murine recombinant TGF-β1 (R&D Systems) in the presence or absence of murine recombinant HGF (R&D Systems) at indicated doses for 24 hr. Cells were then used for evaluation of *α-Sma* expression by real-time PCR and immunohistochemistry.

RNA Isolation and Real-Time qPCR

Total RNA was isolated using the RNeasy Micro Kit (Qiagen). Isolated RNA was reverse transcribed into cDNA using oligo(dT) primer and SuperScript III (Invitrogen). Real-time qPCR was then performed using Taqman Universal PCR Mastermix and pre-formulated Taqman primers for murine glyceraldehyde-3-phosphate dehydrogenase (*Gapdh*), *Hgf*, *Il-10*, *Tsg6*, *Il-1β*, *Tgf-β1*, *Tnf-α*, and *α-Sma* (Life Technologies). The results were analyzed by the comparative threshold cycle method and normalized to *Gapdh* as an internal control.

Immunohistochemistry and Histology

Cryosections of the whole eyeball and fibroblast culture on 8-chamber slides were fixed in acetone and blocked with 2% BSA and anti-FcR antibodies (catalog #14-0161-86, Affymetrix eBioscience). The sections were immunostained with Alexa Fluor 488-conjugated anti-α-SMA or isotype-matched control antibodies (#53-6496-80, Affymetrix) overnight at 4°C. Slides were then mounted using Vector Shield mounting medium (Vector Laboratories) and examined under a confocal microscope. For histological evaluation, corneal sections were stained with H&E and examined using bright-field microscopy.

Flow Cytometry

A single-cell suspension of MSCs was prepared and stained with fluorochrome-conjugated monoclonal antibodies and appropriate isotype controls. Antibodies (Biolegend) against CD45 (catalog #103133), CD34 (#119310), SCA-1 (#108105), CD29 (#102207), and CD105 (#120407) were used for the phenotypic characterization of MSCs. Stained cells were analyzed on an LSR-II flow cytometer (BD Biosciences).

ELISA

Levels of TGF-β1 and HGF in supernatants of MSC cultures or corneal lysates were analyzed using commercially available murine ELISA kits (R&D Systems) as per the manufacturer's instructions.

Statistical Analysis

Mann-Whitney U tests or Student's t tests were performed to determine significance, which was set at $p < 0.05$. Results are presented as the mean ± SD of three independent experiments. In vivo evaluations and quantification of images of corneal injury and opacity were performed in a masked fashion. Samples sizes were estimated on the basis of previous experimental studies on corneal injury and inflammation (Lan et al., 2012; Basu et al., 2014).

SUPPLEMENTAL INFORMATION

Supplemental Information includes three figures and can be found with this article online at http://dx.doi.org/10.1016/j.stemcr.2016.09.001.

AUTHOR CONTRIBUTIONS

S.K.M. and M.O. performed experiments, and contributed to data analysis and manuscript writing. A.A., A.S., A.R., and K.R.K. assisted in performing experiments and data analysis. S.K.S. contributed to manuscript revision and data analysis. D.I.S. assisted in GFP-MSC homing experiments. S.K.C. contributed to the underlying hypothesis, designed the experiments, analyzed data, and wrote the manuscript.

ACKNOWLEDGMENTS

The authors thank Drs. Reza Dana, Balaraj B. Menon and Ahmad Kheirkhah at the Department of Ophthalmology, Harvard Medical School for helpful scientific discussions; and Dr. James Zieske, Schepens Eye Research Institute, Boston for providing human corneal fibroblast cells. The authors would also like to acknowledge the editorial contributions of Drs. Balaraj B. Menon and Susanne Eiglmeier in the preparation of the manuscript. This work was supported in part by grants from the NIH (EY024602 to S.K.C.; P30-EY003790 core grant; and HL131645 to D.I.S.) and the Department Of Defense (W81XWH-15-1-0024 to S.K.C.). Schepens Eye Research Institute has filed for intellectual property rights to technologies derived from this study.

Received: May 31, 2016
Revised: September 1, 2016
Accepted: September 2, 2016
Published: September 29, 2016

REFERENCES

Abed, E., Bouvard, B., Martineau, X., Jouzeau, J.-Y., Reboul, P., and Lajeunesse, D. (2015). Elevated hepatocyte growth factor levels in osteoarthritis osteoblasts contribute to their altered response to bone morphogenetic protein-2 and reduced mineralization capacity. Bone *75*, 111–119.

Basu, S., Hertsenberg, A.J., Funderburgh, M.L., Burrow, M.K., Mann, M.M., Du, Y., Lathrop, K.L., Syed-Picard, F.N., Adams, S.M., Birk, D.E., et al. (2014). Human limbal biopsy-derived stromal stem cells prevent corneal scarring. Sci. Transl. Med. *6*, 266ra172.

Dominici, M., Le Blanc, K., Mueller, I., Slaper-Cortenbach, I., Marini, F., Krause, D., Deans, R., Keating, A., Prockop, D., and Horwitz, E. (2006). Minimal criteria for defining multipotent mesenchymal stromal cells. The International Society for Cellular Therapy position statement. Cytotherapy *8*, 315–317.

Gendron, R.L., Liu, C.Y., Paradis, H., Adams, L.C., and Kao, W.W. (2001). MK/T-1, an immortalized fibroblast cell line derived using cultures of mouse corneal stroma. Mol. Vis. *7*, 107–113.

Hamil, M.B. (2011). Mechanical injury. In Cornea, J.H. Krachmer, M. Mannis, and E.J. Holland, eds. (Mosby | Elsevier), pp. 1169–1185.

Herrero-Fresneda, I., Torras, J., Franquesa, M., Vidal, A., Cruzado, J.M., Lloberas, N., Fillat, C., and Grinyó, J.M. (2006). HGF gene therapy attenuates renal allograft scarring by preventing the profibrotic inflammatory-induced mechanisms. Kidney Int. *70*, 265–274.

Hutcheon, A.E.K., Sippel, K.C., and Zieske, J.D. (2007). Examination of the restoration of epithelial barrier function following superficial keratectomy. Exp. Eye Res. *84*, 32–38.

Jester, J.V. (2008). Corneal crystallins and the development of cellular transparency. Semin. Cell Dev. Biol. *19*, 82–93.

Jester, J.V., Barry-Lane, P.A., Petroll, W.M., Olsen, D.R., and Cavanagh, H.D. (1997). Inhibition of corneal fibrosis by topical application of blocking antibodies to TGF beta in the rabbit. Cornea *16*, 177–187.

Jester, J.V., Brown, D., Pappa, A., and Vasiliou, V. (2012). Myofibroblast differentiation modulates keratocyte crystallin protein expression, concentration, and cellular light scattering. Invest. Ophthalmol. Vis. Sci. *53*, 770–778.

Jiang, Y., Jahagirdar, B.N., Reinhardt, R.L., Schwartz, R.E., Keene, C.D., Ortiz-Gonzalez, X.R., Reyes, M., Lenvik, T., Lund, T., Blackstad, M., et al. (2002). Pluripotency of mesenchymal stem cells derived from adult marrow. Nature *418*, 41–49.

Lan, Y., Kodati, S., Lee, H.S., Omoto, M., Jin, Y., and Chauhan, S.K. (2012). Kinetics and function of mesenchymal stem cells in corneal injury. Invest. Ophthalmol. Vis. Sci. *53*, 3638–3644.

Lee, R.H., Yu, J.M., Foskett, A.M., Peltier, G., Reneau, J.C., Bazhanov, N., Oh, J.Y., and Prockop, D.J. (2014). TSG-6 as a biomarker to predict efficacy of human mesenchymal stem/progenitor cells (hMSCs) in modulating sterile inflammation in vivo. Proc. Natl. Acad. Sci. USA *111*, 16766–16771.

Ljubimov, A.V., and Saghizadeh, M. (2015). Progress in corneal wound healing. Prog. Retin. Eye Res. *49*, 17–45.

Nishida, T., and Saika, S. (2011). Cornea and sclera. In Cornea, J.H. Krachmer, M. Mannis, and E.J. Holland, eds. (Mosby | Elsevier), pp. 3–24.

Omoto, M., Katikireddy, K.R., Rezazadeh, A., Dohlman, T.H., and Chauhan, S.K. (2014). Mesenchymal stem cells home to inflamed ocular surface and suppress allosensitization in corneal transplantation. Invest. Ophthalmol. Vis. Sci. *55*, 6631–6638.

Resnikoff, S., Pascolini, D., Mariotti, S.P., and Pokharel, G.P. (2008). Global magnitude of visual impairment caused by uncorrected refractive errors in 2004. Bull. World Health Organ. *86*, 63–70.

Torricelli, A.A.M., Santhanam, A., Wu, J., Singh, V., and Wilson, S.E. (2016). The corneal fibrosis response to epithelial-stromal injury. Exp. Eye Res. *142*, 110–118.

Uccelli, A., Moretta, L., and Pistoia, V. (2008). Mesenchymal stem cells in health and disease. Nat. Rev. Immunol. *8*, 726–736.

Wang, J., Liao, L., and Tan, J. (2011). Mesenchymal-stem-cell-based experimental and clinical trials: current status and open questions. Expert Opin. Biol. Ther. *11*, 893–909.

Whitcher, J.P., Srinivasan, M., and Upadhyay, M.P. (2001). Corneal blindness: a global perspective. Bull. World Health Organ. *79*, 214–221.

Yi, X., Li, X., Zhou, Y., Ren, S., Wan, W., Feng, G., and Jiang, X. (2014). Hepatocyte growth factor regulates the TGF-β1-induced proliferation, differentiation and secretory function of cardiac fibroblasts. Int. J. Mol. Med. *34*, 381–390.

Stem Cell Reports, Volume 7

Supplemental Information

Restoration of Corneal Transparency by Mesenchymal Stem Cells

Sharad K. Mittal, Masahiro Omoto, Afsaneh Amouzegar, Anuradha Sahu, Alexandra Rezazadeh, Kishore R. Katikireddy, Dhvanit I. Shah, Srikant K. Sahu, and Sunil K. Chauhan

Supplemental Data

<u>Figure S1</u>.

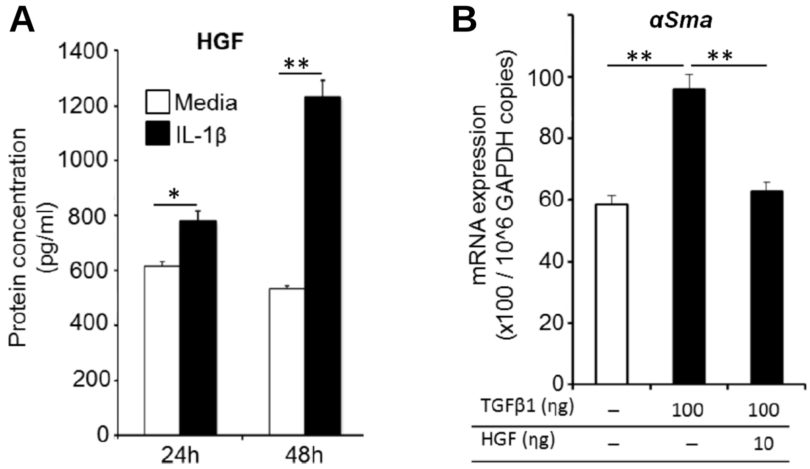

<u>Fig S1</u>: *HGF expressoin by human MSCs, and effect of recombinant HGF on human corneal firoblast.* **[A].** Human MSCs secrete elevated levels of HGF upon IL1β stimulation. Human MSCs (purchased from Lonza) were cultured in medium alone (white bars) or with IL1β (100ng/ml, Peprotech) (black bars) for 24 hrs and 48 hrs. HGF protein levels were estimated in culture supernatants using ELISA (R&D systems). **[B].** HGF inhibits differentiation of human corneal fibroblasts into myofibroblasts. Human corneal fibroblasts (gifted from James Zieske laboratory, Schepens Eye Reaesch Institute) were stimulated with human recombinant TGFβ1 (100ng/ml, Peprotech) in the presence or absence of rhHGF (10ng/ml, R&D Systems) for 24hrs. *α-SMA* expression (normalized to internal control *GAPDH*) was evaluated using real-time PCR.The values shown are mean ± SD (error bars) from three independent experiments performed in triplicates, * p<0.02, ** p<0.001.

<u>Figure S2</u>.

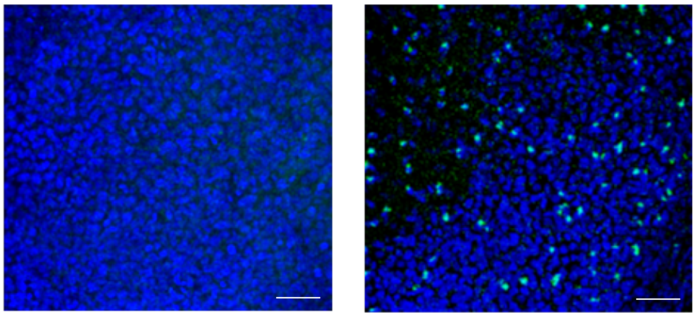

(Blue: DAPI, nuclei | Green: GFP+MSCs)

<u>Fig S2:</u> ***MSCs selectively home to injured corneas.*** Corneal injury was induced by mechanical removal of corneal epithelium and anterior stroma in C57BL/6 mice. After 1 hour of injury, GFP-expressing MSCs purified from GFP transgenic C57BL/6 mice (Jackson Laboratory, Bar Harbor, ME, USA) were intravenously administered to mice (n=5) and corneas were harvested at 7 day post injury. Corneas were fixed with 4% paraformaldehyde for 20 minutes and permeabilized with 0.5% TritonX-100 for 10 minutes, and then immunostained with Alexa Fluor 488-conjugated anti-GFP (Invitrogen). Corneas were then mounted onto slides with DAPI containing mounting medium (Vector Laboratories, Burlingame, CA). Representative confocal micrographs of whole mount corneas showing the presence of GFP+ MSCs (Green color) in the injured cornea, but not in the contralateral (uninjured) cornea (scale bar 100µm).

Figure S3.

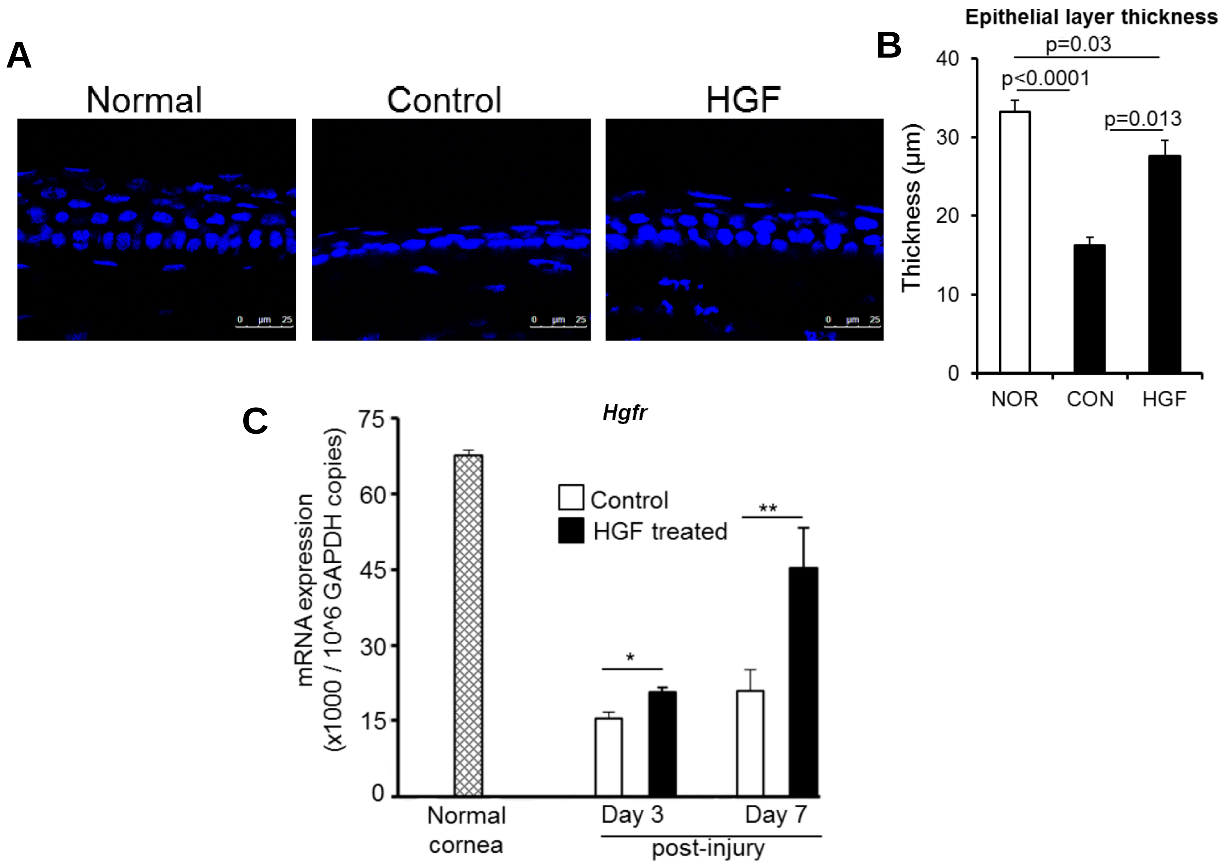

Fig S3: ***HGF augments epethelial stratification and HGF-R (c-met) expression in the cornea after injury.*** **[A]** At 7 days post injury, corneas were harvested from normal, albumin-treated and HGF-treated mice. Corneal cross sections were stained with the nuclear stain DAPI to visualize corneal epithelial cell layer using confocal microscope (scale bar: 25µm). **[B]**. Bar chart showing the thickness (µm) of the epithelial cell layer in normal (white bar), control-injured and HGF-treated injured corneas (black bar). **[C]**. At 3 and 7 days post injury, corneas were harvested from normal (checked bar), mouse albumin-treated control injured (white bar) and HGF-treated (black bar) injured groups. Total RNA was isolated from harvested corneas. *Hgf-R* mRNA expression was quantitated using real-time PCR. *Gapdh* was used as a internal control.The values shown are the mean ± SD; n=5-6 mice/group.
